# Value of 18F-FDG PET/CT Scans in Staging and Follow-Up of Pediatric Langerhans Cell Histiocytosis: Comparison to CT and/or MRI

**DOI:** 10.3390/children12081089

**Published:** 2025-08-20

**Authors:** Maria F. Dien Esquivel, Abdullah AlMutawa, Afsaneh Amirabadi, Sheila Weitzman, Ilia Buhtoiarov, Andrea S. Doria, Amer Shammas, Oussama Abla, Reza Vali

**Affiliations:** 1 Department of Diagnostic and Interventional Radiology, The Hospital for Sick Children, Toronto, ON M5G 1X8, Canada; maria.dienesquivel@sickkids.ca (M.F.D.E.);; 2 Department of Medical Imaging, University of Toronto, 263 McCaul St 4th Floor, Toronto, ON M5T 1W7, Canada; 3 Division of Hematology/Oncology, The Hospital for Sick Children, Toronto, ON M5G 1X8, Canada; 4 Department of Pediatric Hematology Oncology and Blood and Marrow Transplantation, Cleveland Clinic Children’s Hospital, Cleveland, OH 44195, USA

**Keywords:** 18F-FDG PET/CT, pediatric imaging, Langerhans cell histiocytosis, children, MRI

## Abstract

**Highlights:**

**What are the main findings?**

• 18F-FDG PET/CT provides diagnostic findings comparable to CT or MRI, with added metabolic information and reduced radiation dose compared to CT.
• 18F-FDG PET/CT shows earlier interval changes after therapeutic intervention compared to CT and MRI.

**What is the implication of the main finding?**

• 18F-FDG PET/CT can identify additional Langerhans cell histiocytosis (LCH) lesions compared to CT and MRI, which is crucial for disease staging and treatment planning.
• 18F-FDG PET/CT is particularly useful in cases of suspected LCH recurrence and for assessment of response to therapy.

**Abstract:**

**Background/Objectives**: The purpose of this study is to determine the added value of 18F-FDG PET/CT scan in pediatric LCH compared to other imaging modalities (CT and MRI) at initial staging, during assessment of disease reactivation, and after treatment. **Methods:** This is a retrospective study of children diagnosed with LCH between 1 June 2007 and 8 December 2022 who met the inclusion criteria. 18F-FDG PET CT imaging was compared to CT and/or MRI when available. The interclass correlation coefficient (ICC) was used to assess the agreement between methods. *p*-Values of less than 0.05 were considered statistically significant. **Results:** A total of 39 children had undergone 18F-FDG PET/CT studies. Median (range) age at presentation was 10 years (1.3–17 y), with a female-to-male ratio of 0.7:1. Excellent concordance (ICC = 1; *p* < 0.0001) between 18F-FDG PET/CT and other imaging methods was found. Median SUVmax of the positive FDG-avid lesions at initial staging was 2.7 [range 1.3–16.7]. **Conclusions:** 18F-FDG PET/CT has been shown to be complementary to diagnostic CT and MRI, with the advantage of demonstrating additional metabolic information at initial staging, during assessment of disease reactivation, and to assess interval changes post therapy. These preliminary findings warrant further investigation.

## 1. Introduction

Langerhans cell histiocytosis (LCH) is a rare, clinically heterogeneous disease affecting predominantly the inflammatory myeloid neoplasm and is caused by activating mutations in the pediatric population, with more prevalence in males [1,2,3]. The diagnosis is histological, and much about its etiology is still unknown [2,4]. LCH can affect any organ or system; however, in children it affects the MAPK (Mitogen-Activated Protein Kinase) pathway, most commonly *BRAF-V600E* (B-Raf proto-oncogene, serine/threonine kinase V600E mutation) and *MAP2K1*. Both children and adults can be affected, with heterogeneous clinical presentations ranging from self-limited to multisystem (MS) life-threatening forms. LCH is characterized by the accumulation of CD1a +/CD207 + (Cluster of Differentiation) cells in different organs and systems, most commonly the bones, skin, and lungs [1,5]. Patients with MS disease involving the liver, spleen, or hematopoietic system have a higher risk of mortality [6].

Classification of LCH depends on the number of lesions and sites of involvement. Single-system LCH involves only one system or organ, with the most commonly affected being the bones and skin [1]. Multisystem LCH affects two or more organs or systems. The disease prognosis and response to therapy will depend on the involvement of high-risk organs or systems such as the liver, spleen, or hematopoietic system [2,3,4,5].

Accurate assessment of pediatric LCH at diagnosis and upon evaluation of early response to therapy is crucial to determining the most appropriate treatment strategy and to allowing early escalation of therapy in those with poor response [4].

Assessment of early response using conventional imaging modalities such as contrast-enhanced CT and MRI, plain radiographs, or CT scans, particularly with skeletal lesions, may be challenging, as normalization of these lesions may take a long time (the earliest evidence of bone healing could be seen after 6 weeks of therapy) [7]. 18F-FDG PET/CT has proven to be more sensitive than conventional radiography or CT in detecting LCH lesions at initial staging and follow-up, except for lung LCH [8]. While MRI has shown to be overall more sensitive than PET in assessing brain lesions [9], previous studies have also shown that 18F-FDG PET/CT is more accurate in evaluating disease activity after chemotherapy [3,5,10,11].

The purpose of this study is two-fold: (1) to determine the added value of 18F-FDG PET/CT in detecting additional lesions at initial staging workup compared to other modalities and (2) to report the usefulness of 18F-FDG PET/CT for interval changes in the regions of interest following therapy compared with clinical outcomes and imaging findings with CT and/or MRI.

## 2. Materials and Methods

### 2.1. Study Population

A research ethics board (REB) approved this retrospective study. Patients aged 0–17 years with clinical and/or histological diagnosis of LCH were included. Children in this study underwent 18F-FDG PET/CT at initial diagnostic workup and/or after response to therapy between 1 June 2007 and 8 December 2022. The institutional research ethics board granted a waiver of consent given the retrospective nature.

### 2.2. Imaging Acquisition

All 18F-FDG PET/CT examinations were obtained according to the standard protocol of our institution (The Hospital for Sick Children) using a (64-multidetector CT) PET–CT hybrid scanner (Discovery VCT 64 Slice; GE Healthcare, Boston, MA, USA). For all patients, weight, height, and blood glucose levels were recorded. Blood glucose concentrations were <11 mmol/L in all patients before FDG administration. Approximately 60 min after injection of 5.18 MBq/kg (0.14 mCi/kg) 18F-FDG, images were acquired for the whole-body PET scan. Doses ranged from 37 MBq (1 mCi) to 370 MBq (10 mCi). Patients were scanned from the vertex of the skull to the feet (3 min per bed position, with an average of 7–10 bed positions per scan). Patients with a recent diagnostic CT were imaged using a reduced-dose helical CT scan (5 mm/slice, 90 kVp; 20 and 30 mAs for patients weighing <30 and ≥30 kg, respectively) prior to the PET scan for attenuation correction and anatomical localization. Diagnostic CT scans were obtained when clinically indicated and when patients did not have a recent diagnostic CT scan. In those cases, the attenuation correction was calculated based on the correlative diagnostic CT images (5 mm slice, 120 kV, and a weight-based range for the mA, with a maximum of 200 mA with dose modulation). Reconstruction of the PET images was performed using the iterative method of line of response (line of response row action maximum-likelihood algorithm or 3-D row action maximum-likelihood algorithm).

### 2.3. Imaging Analysis

One physician with specialties in both pediatric radiology and pediatric nuclear medicine (M.D.E.) reviewed the images without knowing the result of the previous reports. The reports were completed by two pediatric nuclear medicine physicians (R.V. and A.S.) with more than 10 and 15 years of experience after pediatric nuclear medicine fellowships. Discrepant or equivocal findings were reviewed with one pediatric radiologist (A.D.) and one pediatric nuclear medicine physician (R.V.) for a consensus result.

18F-FDG PET/CT scans, CT, and MRI were reviewed when available, and the number of scans; the date of 18F-FDG PET/CT, CT, and/or MRI; background liver and mediastinal blood pool metabolic activity; the number and location of hypermetabolic lesions on 18F-FDG PET/CT; the maximum standardized uptake value (SUV max); the number and location of the target lesions on CT and MRI; and the size of the lesions were collected. Size was calculated on CT or MRI by measuring the maximum diameter of the target lesion in the transverse and/or anteroposterior dimension in the axial projection. Measurement of lesions on MR is usually performed where the lesion is more visible, which is most often in the post-contrast T1 weighted images or T2 weighted images when there is no contrast enhancement, particularly in lesions post treatment changes.

Increased 18FDG uptake (greater than background liver activity) not explained by a normal variant or other pathology (such as infection, inflammation, or benign tumors) was considered a positive lesion on 18F-FDG PET/CT [3,10,11]. The SUVmax values of the areas of increased activity were obtained by drawing a region of interest (ROI) over the lesions in the axial image using the standard software supplied by the vendor. Patients’ charts were reviewed by a pediatric oncology fellow (A.A.).

The hypermetabolic lesions on FDG were compared with CT and MRI when available. CT- or MRI-positive lesions were identified using morphologic criteria including lytic bone lesions, bone irregularity, or heterogeneity on CT; abnormal signal intensity on MRI; or restricted diffusion or enhancement on MRI [3,10]. Lymph nodes ≥10 mm in short diameter were considered positive on CT or MRI [12]. CT or MRI were only included for comparison if they were performed less than one month before or after the 18F-FDG PET/CT. The CT portion of the 18F-FDG PET/CT was included for comparison when there was no diagnostic CT or MRI available. Some of the 18F-FDG PET/CTs included only low-dose CT. The entire body was included in low-dose CT and whole-body MRI. A region of interest was assessed for diagnostic (normal-dose) CT and dedicated structural/diffusion-weighted MRI (for example dedicated chest or abdomen CT/MRI). The accuracy did not suffer due to lack of iodine-based contrast.

The clinical response assessment and state of the disease was evaluated based of the Histiocyte Society criteria [13]. Non-active disease (NAD) was considered when all lesions and symptoms were resolved. On the other hand, active disease (AD) was further classified as better (regression), intermediate (stable or mixed response), or worse (progression) [13]. The 5-point Deauville score criteria were assessed on PET/CT at initial staging, and after 6 weeks of treatment Deauville criteria scores (DSs) of 1–3 were considered complete metabolic response, and DSs of 4 and 5 were considered inadequate response (partial response/stable disease) or progressive disease [14].

### 2.4. Clinical Data

Clinical chart review was performed by a pediatric nuclear medicine fellow (M.D.E.) and a pediatric oncology fellow (A.A.). Age and sex were recorded. Clinical notes at diagnosis and follow-up, treatment, and biopsy results were included. Clinical variables included site of the disease (i.e., bone, skin, lymph nodes), single system or multisystem disease, treatment strategies, and response to therapy.

### 2.5. Statistical Analysis

Demographic and radiological characteristics were summarized descriptively with mean and standard deviation (SD) or median (range) for continuous variables, where appropriate, and dichotomous and count variables were expressed as frequencies and percentages. The Shapiro–Wilk test was performed to assess whether the data were normally distributed. The interclass correlation coefficient (ICC) was used to assess the agreement between methods. *p*-Values of less than 0.05 were considered statistically significant. All statistical analysis was performed using Posit team (2024). RStudio: Integrated Development Environment for R. Posit Software (Version 2024), PBC, Boston, MA, USA. URL http://www.posit.co/ [15].

## 3. Results

Thirty-nine pediatric patients with a diagnosis of LCH who underwent 18F-FDG PET/CT were identified between 1 June 2007 and 8 December 2022. There were 22 males and 17 females, with a median (range) age of 10 y (1.3–17 y). The diagnosis of LCH was histologically confirmed at our institution in 37 (95%) of our cohort patients. In the two remaining patients, the histological diagnosis and initial treatment were carried out in a different country but were stated in the clinical chart. Demographics and clinical characteristics are shown in Table 1.

Twenty-six (67%) patients underwent a single 18F-FDG PET/CT study, twelve (31%) patients had between two and five scans, and one (2%) patient underwent a total of seven studies. Sixteen (41%) studies were performed at initial diagnostic workup, twenty-three (59%) studies were conducted to assess for disease reactivation or progression, and thirty-four (87%) studies were acquired to assess response to treatment or follow-up.

Thirty-one MRIs and fifty-two diagnostic CTs were available for comparison. The average time between MRI and 18F-FDG PET/CT was 15.2 days (±8.92 SD), and between diagnostic CT and 18F-FDG PET/CT PET/CT was 1.5 days [range 0–30 days]. Average clinical follow-up was 44.7 months from diagnosis (±35.4 SD).

### 3.1. Initial Staging

#### 3.1.1. Identification of LCH Lesions at Diagnosis by PET Scans

There was excellent agreement (ICC = 1; *p* < 0.0001) between 18F-FDG PET/CT and CT or MRI when considering the entire body for low-dose CT and considering a region of interest (ROI) for normal-dose CT and dedicated structural and diffusion-weighted MRI.

PET/CT findings at initial staging are detailed in Table S1 (Supplementary Materials). The median SUVmax was 2.7 [range 1.3–16.7] for hypermetabolic lesions. 18F-FDG PET/CT showed increased 18FDG avidity in all LCH lesions in the skull, facial bones, and extremities at initial staging diagnosed by conventional imaging (including whole-body low-dose CT, region-of-interest normal-dose CT, and dedicated structural and diffusion-weighted MRI). LCH lesions in the spine vertebrae, including single or multiple lesions and at different levels (cervical, thoracic, and lumbar), were identified in all cases by 18F-FDG PET/CT except for one patient with multiple small (<8 mm) osteolytic lesions in the thoracic and lumbar vertebrae on diagnostic CT.

**Figure 1:**
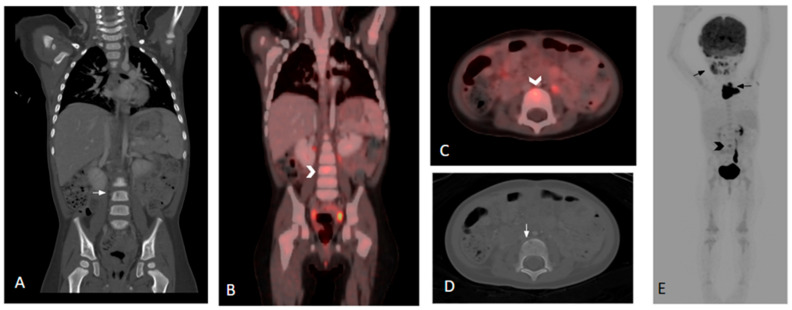
Three-year-old female with histologic diagnosis of LCH. (**A**,**D**) Coronal and axial CT images show an initially missed small lytic lesion at L4 measuring 4.5 mm (white arrows). (**B**,**C**) Fused and whole-body 18F-FDG PET/CT images demonstrate increased FDG activity in the L4 lytic lesion (black and white chevrons). (**E**) Additional lesions in skull, mandible, and thymus are noted in the whole-body 18F-FDG PET/CT (black arrows).

18F-FDG PET/CT proved to be superior to CT in detecting diffuse or multifocal bone marrow involvement in two (5%) patients [both patients had low-dose CT of the extremities and one (2%) had diagnostic CT of the thoracolumbar spine]. 18F-FDG PET/CT was able to identify additional lesions in three (8%) patients compared to conventional imaging: one in the left femur [versus low dose CT], one in the T8 vertebra [versus diagnostic CT] (Figure 1), and one in the L4 vertebra [versus lumbar spine MR]. 18F-FDGPET/CT failed to demonstrate increased FDG avidity in two (5%) patients with pelvic bone LCH lesions identified by diagnostic CT: one with a small lytic lesion in the left acetabulum and one in the left pubic ramus. Both patients were treated for multifocal bone disease.

The 18F-FDG PET/CT scan showed increased FDG avidity in the left tibia in one (2%) patient, but there was no correlation with the low-dose CT, and the follow-up imaging with skeletal survey and WB-MRI did not demonstrate evidence of a bone lesion. However, this did not affect the patient’s treatment plan, as the patient had multifocal bone disease. In another patient, MRI showed an equivocal hyperintense left femoral lesion with mild enhancement but without an abnormality on CT or hypermetabolic lesions on 18F-FDG PET/CT. This patient was treated for multifocal bone disease, with 18F-FDG PET/CT-positive lesions in the left iliac bone (also identified on MRI), T8 vertebra, and left humerus. Follow-up 18F-FDG PET/CT again demonstrated no activity in the left femur (no follow-up MRI was available). 18F-FDG PET/CT and diagnostic CT were concordant in all cases with LCH involvement in the lymph nodes, thymus, spleen, and parotid gland.

#### 3.1.2. Re-Staging or Assessment of Disease Reactivation by PET Scans

There was excellent agreement (ICC = 1) between 18F-FDG PET/CT and CT or MRI when considering low-dose CT for the entire body (*p* = 1) or whole-body MRI (*p* < 0.0001) and considering a region of interest (ROI) for normal-dose CT (*p* < 0.0001) and dedicated structural and diffusion-weighted MRI (*p* < 0.0001) (see Table 2).

18F-FDG PET/CT findings at the assessment of disease reactivation or progression are detailed in Table S2 (Supplementary Materials). The median SUVmax of lesions suspicious for LCH was 3.85 [range 0.8–10.5].

Hypermetabolic activity was detected in LCH lesions in the left parietal bone (*n* = 1), right iliac bone (*n* = 2), left acetabulum (*n* = 2), right clavicle (*n* = 1), left sacroiliac joint (*n* = 1), right iliac bone (*n* = 1), occipital bone (*n* = 1), C5 vertebral body (*n* = 1), right orbit (*n* = 1), left scapula (*n* = 1), and left femur (*n* = 1), and the findings were concordant with conventional imaging (CT or MRI).

All cases with LCH involvement of the lymph nodes, thymus, and liver were also concordant between 18F-FDG PET/CT and diagnostic CT. 18F-FDG PET/CT was able to detect additional lesions in three patients compared to CT and MRI, including the ribs (*n* = 2), T3 vertebral body (*n* = 1), right iliac bone (*n* = 1), and right femur (*n* = 1).

One patient showed increased focal FDG avidity in the bowel (false positive); however, no abnormality was seen on diagnostic CT, and biopsy was negative for LCH.

18F-FDG PET/CT was negative in 19 residual osseous lesions shown on CT or MR ((*n* = 5) in the skull, (*n* = 2) in the cervical spine, (*n* = 1) in the rib, (*n* = 1) in the lung, (*n* = 1) in the shoulder, (*n* = 1) in the femur, and (*n* = 8) in the pelvic bones), which is consistent with the treatment effect and dynamics of therapy-related changes seen in 18F-FDG PET/CT (detects disease activity) and CT and/or MRI (demonstrate bone remodeling and healing).

MRI is the gold standard to investigate CNS involvement. In our study, MRI was useful in identifying cerebral white matter signal changes and pituitary gland abnormalities, with no corresponding areas of increased or decreased metabolism on 18F-FDG PET/CT. Only in one case did PET/CT show increased FDG avidity, in the sella turcica.

Lung involvement was only seen in 2/39 patients in our cohort (diffuse reticulonodular pattern and cystic lung disease on diagnostic CT). None of these patients showed increased hypermetabolism of the lungs on 18F-FDG PET/CT.

Two patients had positive lesions shown by 18F-FDG PET/CT and CT, but they were not visible on MRI: left frontal bone [dedicated MRI head], left orbit, maxillary sinuses, mandible, right scapula, and thoracolumbar spine [whole-body MRI].

#### 3.1.3. Assessment of Response to Therapy by PET Scans

Eleven patients had 18F-FDG PET/CT scans at initial staging or re-staging and after 6 weeks of treatment to assess the response to therapy (Table 3). Two patients had complete metabolic response (Deauville 1–2), seven patients had partial metabolic response (Deauville 3–4), one patient had stable disease (Deauville 5), and one patient had progression of the disease (Deauville 5).

## 4. Discussion

In this manuscript, we provide an outline of 18F-FDG PET/CT findings in children with LCH and attempt to explore the comparison with conventional imaging methods such as CT and MRI. Overall, our results support what has been described in the literature: 18F-FDG PET/CT is an effective diagnostic tool to diagnose and follow up with children with Langerhans cell histiocytosis, with the advantage of evaluating metabolic disease activity, which cannot be assessed with other imaging methods and is particularly useful for detecting disease activity post therapy [3,10,16,17,18].

Several studies have previously shown that 18F-FDG-PET/CT has high sensitivity in detecting active LCH lesions, and the rate of false positives is quite low [10,19]. In our study, 18F-FDG PET/CT was able to identify additional lesions in three patients compared to conventional imaging, and we found only one positive skeletal lesion that resulted in a false positive on follow-up imaging.

18F-FDG PET/CT showed high sensitivity in detecting bone marrow involvement and in the detection of additional skeletal lesions in three patients compared to CT and dedicated MRI, with two in the vertebrae and one in the left femur. This is concordant with previous reports that have shown higher sensitivity of PET/CT compared to conventional imaging in detecting bone involvement [8,11,18,20].

Previous authors have described less sensitivity of 18F-FDG PET/CT than MRI in the detection of spinal lesions [3,16]. In our study, 18F-FDG PET/CT was superior to diagnostic CT and MRI in the detection of spinal lesions in two cases. However, in one case, diagnostic CT was superior to 18F-FDG PET/CT in demonstrating vertebral lesions, which is likely related to the size of those lesions.

**Figure 2:**
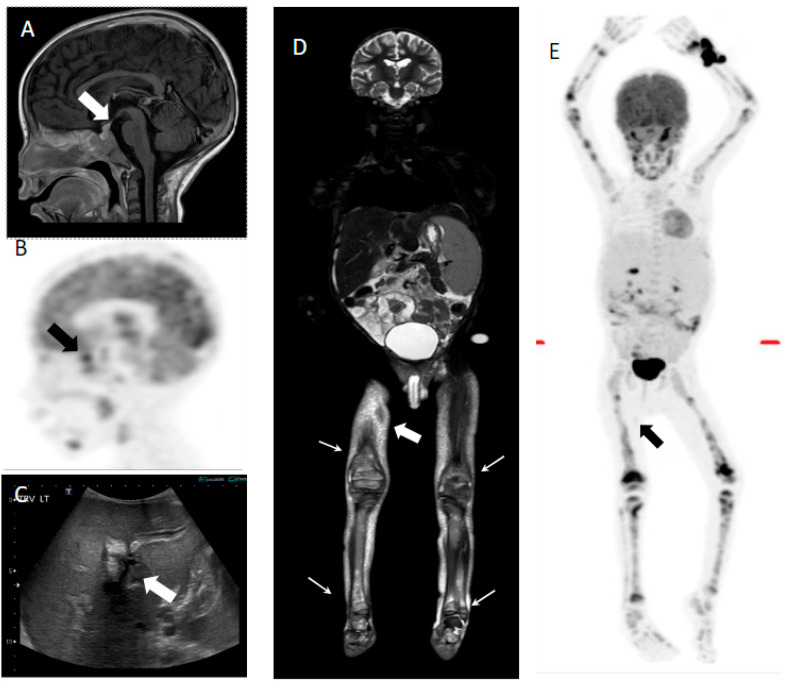
Seven-year-old male with multisystem LCH. (**A**) Sagittal T1 post-contrast image shows thickening and enhancement of the pituitary stalk (white arrow). (**B**) FDG PET shows increased uptake in the sellar region (black arrow). (**C**) Transverse ultrasound image shows hepatomegaly and increased periportal echogenicity/edema (white arrow). (**E**) Whole-body coronal STIR MRI image shows hepatosplenomegaly, ascites, multifocal areas of increased signal in the bone marrow (white arrows), and cutaneous involvement (white filled arrow). (**D**) MIP PET image demonstrates multifocal avid FDG disease involving the axial and appendicular skeleton. Mild focus of uptake is seen in the right mid-thigh, corresponding to a subcutaneous lesion (black arrow). Biopsy of the skin lesions was positive for LCH.

It has been established that diagnostic, high-resolution CT is better for the evaluation of lung involvement [3], and that dedicated brain MRI is superior to 18F-FDG PET/CT for the diagnosis and follow-up of CNS involvement [17]. This was also comparable with our study, except in one case, where 18F-FDG PET/CT was able to demonstrate increased FDG uptake in the sella turcica (Figure 2).

**Figure 3:**
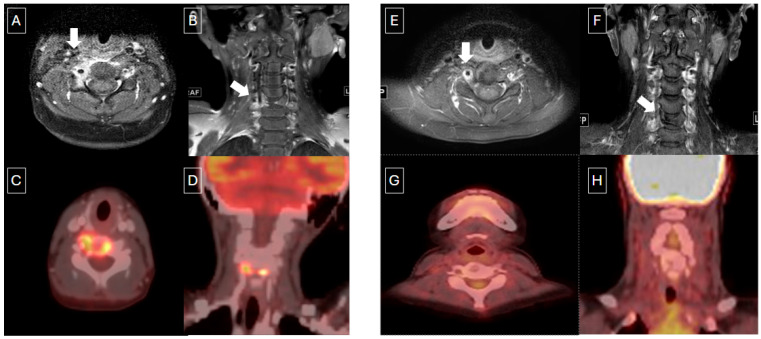
Nine-year-old male with multifocal bone LCH. (**A**,**B**) Axial and coronal T1 post-contrast MR images show vertebra plana and soft tissue enhancement (white arrows) with corresponding increased metabolic activity on PET/CT (**C**,**D**). End-of-therapy assessment on MR (**E**,**F**) showed some enhancement in the soft tissues and persistent vertebra plana. However, post-treatment FDG PET/CT (**G**,**H**) demonstrated complete metabolic response.

18F-FDG PET/CT has shown to be superior to CT and MRI in assessing the response to therapy in patients with LCH [11]. Baratto et al. [21] also described similar accuracy for 18F-FDG PET and WB DW-MRI for staging and therapy response evaluation. In our study, the metabolic response to therapy on 18F-FDG PET/CT was identified earlier than the morphological changes on CT or MRI, and it was concordant with the clinical assessment shown in Figure 3. In our article, we also included response assessment of 18F-FDG PET/CT using Deauville criteria and compared the response with clinical assessment. In patients who were seen via 18F-FDG PET/CT to have had complete metabolic response 6 weeks after treatment, there was no evidence of recurrence on clinical follow-up.

There are several limitations to our study. First is the retrospective nature of the study. Second, not all the suspected LCH lesions were histologically confirmed. Third is the accurate measurement of small lesions of less than 10 mm. This is a challenging scenario and requires specific thresholding and perhaps a specific method to be defined for further studies. Finally, our cohort is relatively small, and because there was long-term data acquisition, not all patients had 18F-FDG PET/CT performed at the same periods (i.e., initial staging and post therapy).

## 5. Conclusions

Our findings suggest that 18F-FDG PET/CT diagnostic CT and MR are complimentary in the diagnosis of pediatric LCH. 18F-FDG PET/CT is particularly useful in cases of suspected LCH recurrence and for assessment of response to therapy. Large prospective trials and larger cohorts are required to further demonstrate the benefit of 18F-FDG PET/CT in pediatric LCH.

## Figures and Tables

**Table 1:** Patient demographics and clinical characteristics.

Patient	Age	Sex	Disease Sites	Timing of PET at Diagnosis	Treatments	Disease Reactivation During Time of Follow-Up
1	11.8	M	Oral cavity and gums	2 months after diagnosis	Observation. No treatment	No
2	14.6	F	Focal bone (tibia) + CNS (DI)	No initial staging PET	CTx	Yes
3	5.2	M	Skull + CNS (DI) reactivation	No initial staging PET	CTx	Yes
4	17	F	Mastoid	No initial staging PET	CTx	Yes
5	5.2	F	Mastoid	No initial staging PET	CTx	No
6	12.10	F	Temporal bone	No initial staging PET	Surgical (curettage)	No
7	3.6	M	Multisystem [skin, MFB, LNs, CNS (ND)]	No initial staging PET	Inhibitors (dabrafenib)	Yes
8	5.2	F	Skull + CNS (ND)	At time of diagnosis	CTx + inhibitors (dabrafenib)	Yes
9	13.7	M	Multisystem [skin, lungs, CNS (ND)]	No initial staging PET	CTx	Yes
10	12.8	F	MFB	No initial staging PET	CTx +indomethacin	Yes
11	2.4	M	Multisystem (skin, BM, liver, spleen, CNS)	4 months after diagnosis	CTx	No
12	2.5	M	LNs	2 months after diagnosis	CTx	No
13	6	M	Multisystem (liver, LNs)	No initial staging PET	CTx + inhibitors (dabrafenib + trametinib) + 2 liver transplants	Yes
14	1.3	F	Multisystem (skin, GI, ear)	At time of diagnosis	CTx	No
15	12.11	M	MFB	No initial staging PET	CTx	Yes
16	12.9	F	Multisystem (MFB, spleen, bowel, BM, CNS)	At time of diagnosis	CTx	Yes
17	12.3	M	MFB	No initial staging PET	CTx + indomethacin	Yes
18	6.4	M	Thoracic spine (T10)	No initial staging PET	CTx	No
19	8.2	M	MFB	No initial staging PET	Indomethacin + CTx	Yes
20	10.1	M	Multisystem (skin, bone, CNS)	No initial staging PET	CTx	Yes
21	6.10	M	Thoracic spine (T10)	No initial staging PET	CTx	No
22	16	F	MFB	No initial staging PET	CTx + indomethacin	Yes
23	11	F	Cervical spine (C3)	At time of diagnosis	CTx + indomethacin	Yes
24	14	F	Left humerus and pituitary gland	No initial staging PET	CTx + indomethacin	Yes
25	16	M	Multisystem (MFB and pituitary)	No initial staging PET	CTx	No
26	8	M	CNS (neurodegenerative) + bone (right orbit)	No initial staging PET	Inhibitors (trametinib + dabrafenib)	Yes
27	7	M	Multisystem (bone, LNs)	At time of diagnosis	CTx + hydroxiurea	Yes
28	9	M	MFB + CNS (neurodegenerative)	At time of diagnosis	CTx + inhibitors (dabrafenib)	Yes
29	15	F	Bone (left femur)	No initial staging PET	CTx + indomethacin	Yes
30	17	F	CNS (pituitary)	No initial staging PET	No treatment at SK, diagnosed in India	No
31	7	M	Multisystem (MFB, liver, skin, pituitary)	1 month after diagnosis	CTx + inhibitors (dabrafenib)	Yes
32	3	M	MFB	At time of diagnosis	CTx	No
33	14	F	MFB + skin	No initial staging PET	Indomethacin + CTx	Yes
34	3	M	Multisystem (scalp, buccal mucosa, skin, LNs, lungs, thymus)	No initial staging PET	CTx + inhibitors (dabrafenib)	Yes
35	14	M	Bone (right tibia)	At time of diagnosis	CTx	No
36	3	F	Multisystem (skin, bones, thymus, LNs)	No initial staging PET	CTx + inhibitors (dabrafenib)	Yes
37	17	M	Bone (lumbar spine)	At time of diagnosis	CTx	No
38	1.3	F	Multisystem (MFB, salivary glands, lungs, LNs)	At time of diagnosis	CTx + indomethacin	Yes
39	10	F	MFB	At time of diagnosis	CTx	No

Abbreviations: CNS = central nervous system; DI = diabetes insipidus; ND = neurodegenerative; MFB = multifocal bone; GI = gastrointestinal; BM = bone marrow; LNs = lymph nodes; CTx = chemotherapy.

**Table 2:** Agreement between PET/CT and CT or MRI.

**Initial Staging**		
	ICC	*p* value
PET/CT vs. LDCT	1	<0.0001
PET/CT ROI vs. CT ROI	1	<0.0001
PET/CT ROI vs. MRI ROI	0.997	<0.0001
**Disease Reactivation Assessment**		
PET/CT vs. LDCT	1	1
PET/CT ROI vs. CT ROI	1	<0.0001
PET/CT ROI vs. MRI ROI	1	<0.0001
PET/CT WB vs. MRI WB	1	<0.0001

Abbreviations: LDCT = low-dose CT; ROI = region of interest; WB = whole body; ICC = interclass correlation coefficient.

**Table 3:** PET-CT findings in treated LCH patients (Deauville criteria): assessment of disease response.

Patient	PET/CT #1 (SUV Max)	Background SUVs #1	PET/CT *n* #2 (SUV Max)	Background SUVs #2	Deauville Score	Status at Follow-Up
13	(Re-staging) Focal activity in the liver, segment 4B (3.4) Right abdomen small bowel uptake (4.1)	Liver 2.0 Mediastinum 1.3	(9 mo post) No hypermetabolic lesions	Liver 2.5 Mediastinum 1.3	1 Complete response	No evidence of recurrence in 2nd transplanted liver
15	(Re-staging) Left parietal bone (5.7) Left 7th rib (7.8) Right iliac crest (2.2) Left acetabulum (6.1) Right level IIb lymph node (2.5) No uptake on C3/C4 vertebrae	Liver 1.4 Mediastinum 1.1	(5 mo post) Left parietal bone (1.5) Left 7th rib (0.9) Right iliac crest (1.8) Left acetabulum (2.3) Right level IIb lymph node (0.8)	Liver 2.0 Mediastinum 1.0	4 Partial response	No evidence of recurrence
16	(At diagnosis) Multiple calvarial lesions, index: right parietal bone (3.4) Right mandibular condyle (3.9) Skull base (5.9) Mid humerus (3.4) Left ribs (1.4) Spleen (6.1) Multiple thoracolumbar spine lesions, index T4 (2.9)	Liver 1.1 Mediastinum 0.7	(3 mo post) Multiple calvarial lesions, index: right parietal bone (*n*/*a*) Right mandibular condyle (2.9) Skull base (*n*/*a*) Mid humerus (2.8) Left ribs (1) Spleen (1.6) Multiple thoracolumbar spine lesions, index T4 (1.6) Right distal tibia (1.1)	Liver 1.4 Mediastinum 1.0	4 Partial response	Clinical evidence of recurrence in different areas, including the brain, one year after treatment
19	(Re-staging) Occipital bone (6.6) Right level II lymph node (2.3)	Liver 3.1 Mediastinum 2.0	(2.5 mo post) Persistent uptake in the occipital bone lesion (6.0) and right level II lymph node (3.0)	Liver 2.6 Mediastinum 1.9	5 Stable disease	After 2 months of indomethacin there was no response to therapy, switched to VBL/Pred + 6MP/MTX
23	(At diagnosis) Right iliac bone (10.5) C5 vertebral body (1.5) Cervical lymph nodes, index: right level IIb (3.3)	Liver 1.8 Mediastinum 1.1	(3 mo post) Mild residual activity in the right iliac bone (2.3) C5 vertebral body (2.2) Cervical lymph nodes, index: right level IIb (2.0)	Liver 2.4 Mediastinum 1.5	4 Partial response	AD better, indomethacin continued for 2 y. Recurrence in different sites (right tibia + LNS)
27	(At diagnosis) Lt iliac bone (5.7) Cervical LNs (3.9)	Liver 1.7 Mediastinum 1.1	(4 mo post) Lt iliac bone (2.3) Cervical LNs (1.8) New right iliac lesion (7.4)	Liver 1.7 Mediastinum 1.3	5 Disease progression	AD better in Lt iliac bone and in cervical LNs, but new LCH in a different area (Rt iliac bone) and considered a relapse; therapy escalated
29	(Re-staging) Left proximal femur uptake (2.2)	Liver 2.2 Mediastinum 1.3	(20.5 mo post) No hypermetabolic lesions	Liver 2.7 Mediastinum 1.7	1 Complete response	NAD Salvage VCR/Ara-C/prednisone then maintenance for 2 years
32	(At diagnosis) Occipital bone (2.9) Lower cervical vertebrae C6 (3.7) Cervical lymph nodes, index: left (3.5) Sternal body (5.0) Right sixth rib (4.6) Axillary lymph nodes (1.3) Left iliac bone (6.1) Right femoral shaft (1.8) Pubic ramus (1.3) No activity in the thoracolumbar vertebrae	Liver 1.3 Mediastinum 1.0	(2.6 mo post) Interval resolution of lesions in the occipital bone, sternal body, right sixth rib, axillary lymph nodes, and pubic ramus Interval improvement in activity in the lower cervical spine (1.7), cervical lymph nodes (2.1), left iliac bone (2.2), and right femoral shaft (1.5) No activity in thoracolumbar spine lesions	Liver 1.8 Mediastinum 1.1	4 Partial response	Better NAD and AD
35	(At diagnosis) Right mid tibia (2.5) Right inguinal lymph node (2.2) No activity in the left pubic tubercle	Liver 2.2 Mediastinum 1.5	(1.8 mo post) Interval improvement in activity in the right mid tibia (1.8) No hypermetabolic inguinal lymph nodes No activity in the left pubic tubercle	Liver 2.0 Mediastinum 1.2	3 Partial response	AD better after 6 weeks of induction NAD at end of treatment after 1 year
36	(Re-staging) Multiple skull lesions, index: right temporal (6.1) Right mandible (5.8) L4 vertebra (4.0) Thymus (9.4)	Liver 1.3 Mediastinum 0.9	(2.5 mo post) Decreased activity in multiple skull lesions, index: right temporal (2.8) Right mandible (5.0) L4 vertebra (2.6) Thymus (4.5)	Liver 1.4 Mediastinum 0.8	4 Partial response	Relapsed LCH BRAF + ve, relapsed in Dec 2020, multiple sites received salvage therapy (VCR AraC pred) for 3 months, then repeated PET/CT in March 2021 showed some improvement and AD was labeled as better. Continued treatment for another 3 months, but at end of April showed signs of progression on follow-up PET/CT
38	(At diagnosis) Multiple skull lesions, index: left orbit (5.9) Cervical lymphadenopathy, index: right (7.7) Salivary glands (7.7) Posterior cervical node (2.3) Axillary lymph nodes (3.9) Left scapula (4.1) Thoracic and lumbar vertebrae, index: T1 (1.3) Pelvic bones, index: right iliac (3.6) Inguinal lymph nodes, index: right (6.4) Right 4th rib (1.8) Upper and lower extremities (2.2) Right popliteal lymph node (1.3)	Liver 1.1 Mediastinum 0.7	(1.4 mo post) Interval improvement in multiple hypermetabolic lesions in the skull (2.7) Cervical lymph nodes, index: left IIa (2.5) Interval improvement in salivary gland activity (1.3) Resolution of activity in the paraspinal lymph node (0.5) Axillary nodes (1.3) Left scapula (1.8) Thoracic and lumbar vertebrae, index: T1 (2.0) Pelvic bones, index: right iliac (3.6) Inguinal nodes (6.4) Right rib uptake (2.0) Upper extremities (1.3) Lower extremities (2.2) Right popliteal lymph node (0.2)	Liver 1.2 Mediastinum 0.7	4 Partial response	AD better 6 weeks after induction. Progressive disease in May 2022 12 weeks after induction required salvage therapy. Progressive disease in July 2022 while on 2-CdA/Ara-C salvage therapy, so treatment shifted to clofarabine. AD better in September 2022.

Abbreviations: NAD—non-active disease; AD—active disease; N/A—not applicable.

## Data Availability

The original contributions presented in the study are included in the article/Supplementary Materials, further inquiries can be directed to the corresponding authors.

## Supplementary Materials

The following supporting information can be downloaded at: https://www.mdpi.com/article/10.3390/children12081089/s1, Table S1. Comparison of PET/CT results with CT and MRI. Table S2. Comparison of PET/CT results with CT and MRI.

## Abbreviations

LCH	Langerhans cell histiocytosis
MRI	Magnetic Resonance Imaging
FDG	Fluorodeoxyglucose
PET	Positron emission tomography
CT	Computer tomography
REB	Research ethics board
ROI	Region of interest
AD	Active disease
NAD	Non-active disease
DS	Deauville Score
ICC	Interclass correlation coefficient
