# Supplementary material for: Value of 18F-FDG PET/CT Scans in Staging and Follow-Up of Pediatric Langerhans Cell Histiocytosis: Comparison to CT and/or MRI
Source: Children (Basel). 2025 Aug 20;12(8):1089. doi: 10.3390/children12081089 (PMC12384199; doi:10.3390/children12081089)
Supplement: Supplementary file 1 [file children-12-01089-s001.zip › children-3769734-supplementary.pdf]

Supplementary Material

Table S1. Comparison of PET/CT results with CT and MRI				
Initial Staging				
Patient	Age/gender	PET/CT (number of lesions)	CT (number of lesions)	MRI (number of lesions)
1	12/M	**[WB]**<br>No hypermetabolic lesions	**[Low dose - WB]**<br>None	N/A
8	5/F	**[WB]**<br>Skull (3)	**[Low dose - WB]**<br>Skull (3)	N/A
8	5/F	**[ROI head]**<br>Skull (3)	**[Low dose - ROI head]**<br>Skull (3)	**[ROI head]**<br>Skull (3)<br>Pituitary (1)<br>White matter signal changes (1)
12	3/M	**[ROI NCAP]**<br>Right parotid gland (1)<br>Bilateral cervical lymph nodes (2)<br>Right paratracheal lymph node (1)	**[Diagnostic ROI NCAP]**<br>Right parotid gland (1)<br>Bilateral cervical lymph nodes (2)<br>Right paratracheal lymph node (1)	N/A
14	1/F	**[ROI head]**<br>External auditory canals (2)	**[Diagnostic ROI Head]**<br>External auditory canals (2)	N/A
16	13/F	**[WB]**<br>Skull (9)<br>Right mandible (1)<br>Sternum (1)<br>Spleen (1)<br>Left 7th rib (1)<br>Multifocal and diffuse bone marrow uptake (19)	**[Low dose - WB]**<br>Skull (9)<br>Right mandible (1)<br>Right humerus (1)<br>Sternum (1)<br>Left 7th-8th ribs (2)<br>Right sixth rib (1)<br>Spleen (1)<br>Multifocal and diffuse bone marrow uptake (11)	N/A
23	11/F	**[ROI cervical spine]**<br>C5 vertebra (1)	**[Diagnostic ROI cervical spine]**<br>C5 vertebral body (1)	**[ROI cervical spine]**<br>C5 vertebral body (1)
25	16/M	**[WB]**<br>Left tibia (1)	**[Low dose - WB]**<br>None	N/A
27	7/M	**[WB]**	**[Low dose - WB]**	N/A

		Left iliac bone (1)<br>Cervical lymph nodes (2)	Left iliac bone (1)<br>Cervical lymph nodes (2)	
27	7/M	**[ROI pelvis]**<br>Left iliac bone (1)	**[Low dose – ROI pelvis]**<br>Left iliac bone (1)	**[ROI pelvis]**<br>Left iliac bone (1)
28	9/M	**[WB]**<br>Cervical spine (2)<br>Left femur (1)	**[Low dose - WB]**<br>Cervical spine (2)	N/A
28	9/M	**[ROI cervical spine]**<br>Cervical spine C1-C2 (2)	**[Low dose -ROI cervical spine]**<br>Cervical spine C1-C2 (2)	**[ROI cervical spine]**<br>Cervical spine C1-C2 (2)
30	17/F	**[WB]**<br>No hypermetabolic lesions	**[Low dose – WB]**<br>None	N/A
30	17/F	**[ROI head]**<br>No hypermetabolic lesions	**[Low dose - ROI head]**<br>None	**[ROI head]**<br>Pituitary (1)
32	3/M	**[ROI Head and Extremities]**<br>Occipital bone (1)<br>Right femur (1)	**[Low dose – ROI head and extremities]**<br>Occipital bone (1)<br>Right femur (1)	N/A
32	3/M	**[ROI NCAP]**<br>Cervical vertebrae (2)<br>Cervical lymph nodes (2)<br>Sternum (1)<br>Right sixth rib (1)<br>Axillary lymph nodes (2)<br>Left iliac bone (1)<br>Pubic ramus (1)	**[Diagnostic ROI NCAP]**<br>Cervical vertebrae (2)<br>Cervical lymph nodes (2)<br>Sternum (1)<br>Right sixth rib (1)<br>Axillary lymph nodes (2)<br>Left iliac bone (1)<br>Pubic ramus (1)<br>Thoracic vertebrae (7)	N/A
35	14/M	**[ROI extremities]**<br>Right tibia (1)	**[Low dose - ROI extremities]**<br>Right tibia (1)	N/A
35	14/M	**[ROI NCAP]**<br>Right inguinal lymph node (1)	**[Diagnostic ROI NCAP]**<br>Right inguinal lymph node (1)<br>Left pubic ramus (1)	N/A
36	3/F	**[ROI head]**<br>Skull (9)<br>Right mandible (1)	**[Low dose – ROI head]**<br>Skull (9)	N/A

			Right mandible (1)	
36	3/F	**[ROI NCAP]**<br>L4 vertebra (1)<br>Thymus (1)	**[Diagnostic ROI NCAP]**<br>L4 vertebrae (1)<br>Thymus (1)	N/A
37	17/M	**[ROI lumbar spine]**<br>Lumbar vertebrae L1, L4 (2)	**[Diagnostic ROI lumbar spine]**<br>Lumbar vertebrae L1, L4 (2)	**[ROI lumbar spine]**<br>Lumbar vertebrae (1)
37	17/M	**[ROI CAP]**<br>None	**[Diagnostic ROI CAP]**<br>Left acetabulum (1)<br>Tiny pulmonary nodules (1)	N/A
37	17/M	**[ROI extremities]**<br>Right femur (1)	**[Low dose- ROI extremities]**<br>Right femur (1)	N/A
38	1/F	**[ROI head and neck]**<br>Skull (15)<br>Left mandible (1)<br>Cervical spine (3)<br>Cervical lymph nodes (2)<br>Salivary glands (2)<br>Posterior cervical node (1)	**[Diagnostic ROI head and neck]**<br>Skull (15)<br>Left mandible (1)<br>Cervical spine (3)<br>Cervical lymph nodes (2)<br>Salivary glands (2)<br>Posterior cervical node (1)	**[ROI head and neck]**<br>Skull (15)<br>Left mandible (1)<br>Cervical lymph nodes (2)<br>Salivary glands (2)<br>Posterior cervical node (1)
38	1/F	**[ROI CAP]**<br>Axillary lymph nodes (2)<br>Thymus (1)<br>Left scapula (1)<br>Diffuse uptake of the thoracic and lumbar spine (5)<br>Pelvic bones (2)<br>Inguinal lymph nodes (2)<br>Right 4th rib (1)	**[Diagnostic ROI CAP]**<br>Axillary lymph nodes (2)<br>Thymus (1)<br>Left scapula (1)<br>Multiple pulmonary lung nodules (1)<br>Thoracic and lumbar vertebrae (4)<br>Pelvic bones (2)<br>Inguinal lymph nodes (2)<br>Right 4th rib (1)	N/A
38	1/F	**[ROI extremities]**<br>Upper and lower extremities (2) diffuse uptake	**[Low dose – ROI extremities]**<br>None	N/A
39	10/F	**[ROI lower extremities]**<br>Left iliac bone (1)	**[Low dose -ROI lower extremities]**<br>Left iliac bone (1)	**[ROI lower extremities]**<br>Left iliac bone (1)

				Left femur (1)
39	10/F	**[ROI NCAP]**<br>T8 vertebra (1)<br>Left humerus (1)	**[Diagnostic- ROI NCAP]**<br>Left humerus (1)	N/A

Abbreviations: NCAP= neck, chest, abdomen and pelvis CAP=chest, abdomen and pelvis WB= whole body ROI= region of interest

**Table S2.** Comparison of PET/CT results with CT and MRI				
Re-staging/Recurrence				
Patient	Age/gender	PET/CT (number of lesions)	CT (number of lesions)	MRI
3	5/M	**[WB]**<br>No hypermetabolic lesions	**[Low dose -WB]**<br>None	N/A
4	17/F	**[WB]**<br>Left axillary lymph node (1)<br>No clinical correlation	**[Low dose -WB]**<br>Left axillary lymph node (1)	N/A
9	14/M	**[ROI head]**<br>No hypermetabolic lesions	**[Diagnostic ROI head]**<br>Skull (1)	**[ROI head]**<br>Skull (1)<br>White matter signal changes (1)
10	13/F	**[ROI NCAP]**<br>No hypermetabolic lesions	**[Diagnostic ROI NCAP]**<br>Right orbit (1)<br>Right 4th rib (1)<br>Right ischium (1)<br>Sacrum (1)	N/A
11	2/M	**[ROI NCAP]**<br>Left mastoid (1)	**[Diagnostic ROI NCAP]**<br>Left mastoid (1)<br>Right orbit (1)<br>Right sphenoid (1)<br>Right lateral sella (1)	N/A
12	3/M	**[ROI NCAP]**<br>Cervical lymph nodes (2)<br>Right paratracheal node (1)	**[Diagnostic ROI NCAP]**<br>Cervical lymph nodes (2)<br>Right paratracheal node (1)	N/A
13	6/M	**[ROI NCAP]**<br>Liver (1)	**[Diagnostic ROI NCAP]**<br>Liver (1)	N/A
15	13/M	**[ROI cervical spine]**	**[Low dose - ROI cervical spine]**	**[ROI cervical spine]**

		None	Cervical spine (2)	Cervical spine (2)
15	13/M	**[WB]**<br>Left parietal bone (1)<br>Left 7th rib (1)<br>Right iliac crest (1)<br>Left acetabulum (1)<br>Right cervical lymph node (1)	**[Low dose -WB]**<br>Left parietal bone (1)<br>Right iliac crest (1)<br>Left acetabulum (1)<br>Right cervical lymph node (1)	N/A
17	12/M	**[WB]**<br>Right clavicle (1)<br>Left SI joint (1)<br>Left acetabulum (1)	**[Low dose -WB]**<br>Right clavicle (1)<br>Left SI joint (1)<br>Left acetabulum (1)	N/A
19 (1)	8/M	**[ROI head and pelvis]**<br>Right iliac bone (1)<br>Left frontal bone (1)	**[Low dose - ROI head and pelvis]**<br>Right iliac bone (1)<br>Left frontal bone (1)	**[ROI head and pelvis]**<br>Right iliac bone (1)<br>Right acetabulum (1)
19 (2)	8/M	**[ROI head]**<br>Occipital bone (1)	**[Diagnostic ROI head]**<br>Occipital bone (1)	**[ROI head]**<br>Occipital bone (1)
19 (2)	8/M	**[ROI neck]**<br>Bilateral cervical lymph nodes (2)	**[Diagnostic ROI neck]**<br>Bilateral cervical lymph nodes (2)	N/A
22	16/F	**[WB]**<br>T3 vertebral body (1)<br>Right 3rd rib (1)<br>Right iliac bone (1)	N/A	**[Whole body MR]**<br>Right shoulder (1)<br>Left upper lobe lung (1)<br>Left iliac crest (1)<br>Left acetabulum (1)<br>Left ilium (1)<br>Left femoral neck (1)
22	16/F	**[ROI chest]**<br>T3 vertebral body (1)<br>Right 3rd rib (1)	**[Diagnostic ROI chest]**<br>Left subpleural lung nodules (1)	N/A
23	11/F	**[ROI pelvis]**<br>Right iliac bone (1)	**[Diagnostic ROI pelvis]**<br>Right iliac bone (1)	**[ROI pelvis]**<br>Right iliac bone (1)
24	14/F	**[ROI NCAP]**<br>Cervical lymph nodes (2)	**[Diagnostic ROI NCAP]**<br>Cervical lymph nodes (2)	N/A
24	14/F	**[ROI head]**<br>None	**[Diagnostic ROI head]**	**[ROI head]**<br>Left parietal bone (1)

			Left parietal bone (1)	Pituitary (1)<br>White matter signal changes (1)
26 (1)	8/M	**[ROI head + orbits]**<br>Right orbit (1)	**[Diagnostic ROI head + orbits]**<br>Right orbit (1)	**[ROI head + orbits]**<br>Right orbit (1)
26 (2)	8/M	**[ROI head + orbits]**<br>No hypermetabolic lesions	**[Diagnostic ROI head + orbits]**<br>None	**[ROI head + orbits]**<br>None
27	7/M	**[WB]**<br>Cervical lymph nodes (2)<br>Left scapula (1)	**[Low dose -WB]**<br>Cervical lymph nodes (2)<br>Left scapula (1)<br>Pelvic bones (2)	N/A
29	15/F	**[WB]**<br>Left femur (1)	**[Low dose -WB]**<br>Left femur (1)	N/A
31	7/M	**[WB]**<br>Left orbit (1)<br>Skull (2)<br>Maxillary sinuses (1)<br>Mandible (1)<br>Right scapula (1)<br>Right iliac bone (1)<br>Right thigh (1)<br>Ribs (2)<br>Multifocal and diffuse bone marrow uptake (12)	**[Low dose -WB]**<br>Left orbit (1)<br>Skull (2)<br>Maxillary sinuses (1)<br>Mandible (1)<br>Right scapula (1)<br>Right iliac bone (1)<br>Right thigh (1)<br>Ribs (2)<br>Multifocal and diffuse bone marrow uptake (12)	**[Whole body MR]**<br>Skull (2)<br>Right iliac bone (1)<br>Right thigh (1)<br>Ribs (1)<br>Multifocal and diffuse bone marrow uptake (10)
31	7/M	**[ROI sella]**<br>Sella (1)	**[Low dose - ROI sella]**<br>Sella (1)	**[ROI sella]**<br>Sella (1)
34	3/M	**[ROI NCAP]**<br>Cervical lymph nodes (2)<br>Thymus (1)	**[Diagnostic ROI NCAP]**<br>Cervical lymph nodes (2)<br>Thymus (1)<br>Lung (1)	N/A
34	3/M	**[ROI extremities]**<br>Right femur (1)	**[Low dose- ROI extremities]**<br>None	N/A

Abbreviations: NCAP= neck, chest, abdomen and pelvis CAP=chest, abdomen and pelvis WB= whole body ROI= region of interest
